# Viral Aetiology of Acute Flaccid Paralysis Surveillance Cases, before and after Vaccine Policy Change from Oral Polio Vaccine to Inactivated Polio Vaccine

**DOI:** 10.1155/2014/814908

**Published:** 2014-03-19

**Authors:** T. S. Saraswathy Subramaniam, Mohd Apandi Apandi, Rohani Jahis, Mohd Samsul Samsudin, Zainah Saat

**Affiliations:** ^1^Virology Unit, Institute for Medical Research, Jalan Pahang, 50588 Kuala Lumpur, Malaysia; ^2^Surveillance Unit, Disease Control Division, Ministry of Health, Level 6, Block E10, 62590 Putrajaya, Malaysia

## Abstract

Since 1992, surveillance for acute flaccid paralysis (AFP) cases was introduced in Malaysia along with the establishment of the National Poliovirus Laboratory at the Institute for Medical Research. In 2008, the Ministry of Health, Malaysia, approved a vaccine policy change from oral polio vaccine to inactivated polio vaccine (IPV). Eight states started using IPV in the Expanded Immunization Programme, followed by the remaining states in January 2010. The objective of this study was to determine the viral aetiology of AFP cases below 15 years of age, before and after vaccine policy change from oral polio vaccine to inactivated polio vaccine. One hundred and seventy-nine enteroviruses were isolated from the 3394 stool specimens investigated between 1992 and December 2012. Fifty-six out of 107 virus isolates were polioviruses and the remaining were non-polio enteroviruses. Since 2009 after the sequential introduction of IPV in the childhood immunization programme, no Sabin polioviruses were isolated from AFP cases. In 2012, the laboratory AFP surveillance was supplemented with environmental surveillance with sewage sampling. Thirteen Sabin polioviruses were also isolated from sewage in the same year, but no vaccine-derived poliovirus was detected during this period.

## 1. Introduction

Poliomyelitis is an acute communicable viral disease affecting humans, mainly young children. The disease is caused by 3 serotypes of Poliovirus (Poliovirus types 1, 2, and 3), belonging to the genera* Enterovirus* and Picornaviridae family. Recently, the virus has been reclassified as* Enterovirus C* spp. in the* Enterovirus* genus [1]. The virus is transmitted through contaminated food and water and multiplies in the intestine, from where it can invade the nervous system. Many infected individuals may be asymptomatic but do excrete the virus in their faeces, hence transmitting infection to others. In about 1% of affected individuals, the virus enters the central nervous system and replicates in anterior horn cells, that is, motor neurons of the spinal cord. The typical neurological manifestation of paralytic poliomyelitis is acute flaccid paralysis (AFP) of limbs, predominantly lower limbs, usually asymmetric and with intact sensation. In rare cases, viral destruction of bulbar cells results in respiratory paralysis and even arrest.

The World Health Assembly in 1988 resolved to eradicate poliomyelitis from the world and marked the launch of the Global Polio Eradication Initiative (GPEI). The GPEI is a partnership led by national governments and spearheaded by the World Health Organization (WHO), Rotary International, the US Centers for Disease Control and Prevention (CDC), and the United Nations Children's Fund (UNICEF) [2]. Since then, there has been a decline in global polio incidence, from an estimated 350,000 cases in 1988 to under 3,500 in the year 2000 [3]. In 2012, a total of 223 polio cases were reported from five countries: Afghanistan, Chad, Niger, Nigeria, and Pakistan. Out of these cases, 97% (217 out of the 223) were reported from the three remaining endemic countries: Afghanistan, Nigeria, and Pakistan [4].

Polio eradication strategies rest on two main activities: immunization coverage and surveillance of acute flaccid paralysis (AFP) cases. Oral polio vaccine (OPV) has been the choice for routine immunization in over 120 countries that have eliminated poliomyelitis [5]. In Malaysia, OPV was licensed and introduced into the childhood immunization program in 1972. Since then, the incidence of poliomyelitis declined with no reported cases between 1986 and 1991. In 1992, Malaysia experienced a small outbreak with 3 cases of paralytic poliomyelitis caused by importation of wild poliovirus that originated from the Indian subcontinent [6]. No wild poliovirus has been identified in Malaysia since 1993.

The OPV consists of live-attenuated virus of the three serotypes. Various studies from developing countries suggest that, after 3 doses of OPV, the mean proportion of infants with detectable serum neutralizing antibodies level was only 73% (36–99%) for type 1, 90% (71–100%) for type 2, and 70% (40–99%) for type 3 poliovirus [7]. This suboptimal seroconversion was related to many factors including interference with other enteroviruses, inhibition of type 1 and type 3 by type 2 in the OPV, diarrheal illnesses, and presence of maternal antibodies [8].

Although OPV is a very safe vaccine, on rare occasions, vaccine-associated paralytic poliomyelitis (VAPP) may occur following ingestion of OPV. The mechanism of VAPP is believed to be a mutation or reversion of the vaccine virus to a more neurotropic form [9]. Vaccine-associated paralytic poliomyelitis is defined by WHO as paralytic poliomyelitis occurring in a vaccinated individual between the 7th and 30th day after receiving a dose or in a close contact of the vaccinated recipient between the 7th and 60th day after a dose was taken [10]. Sabin viruses can replicate in populations with low OPV coverage, acquire the neurovirulence and transmissibility characteristics of WPV, and cause circulating vaccine-derived poliovirus (cVDPV) cases and outbreaks [11, 12]. The ways in which cVDPVs are generated and the conditions that promote them are still not clear. Many cVDPVs have the sequences of non-polio enteroviruses. It is believed that these sequences are acquired by reassortment inside the intestinal tract of an infected person with the OPV virus and the enterovirus; the progression of the infection remains unknown.

In September 2003, the WHO Informal Consultation on identification and management of vaccine-derived polioviruses concluded that, after eradication of wild poliovirus, continued use of OPV would compromise the goal of a polio-free world. IPV could be useful preeradication for prevention of paralysis due to wild or vaccine-derived polioviruses [13]. Most industrialized countries had already decided that, in their specific settings (i.e., geographical distance from endemic countries, very high immunization coverage, temperate climates, and high sanitation and hygiene), the risks of cVDPVs and of VAPP due to continued use of OPV are greater than those due to wild poliovirus importations. Consequently, some of these countries have adopted routine vaccination schedules that rely either exclusively on inactivated polio vaccine (IPV) or on a sequential IPV/OPV schedule [14, 15].

In October 2008, the Ministry of Health, Malaysia, approved a vaccine policy change from OPV to IPV. The switch to IPV in Malaysia into the Expanded Programme of Immunization was rolled out in 8 states: Selangor, Federal Territory, Sabah (including Labuan) Sarawak, Perak, Pahang, Kelantan, and Terengganu. In January 2010, all states used IPV in the EPI. OPV is still used as booster immunization for seven year olds at school entry.

Malaysia has been free of indigenous transmission of wild poliovirus circulation since 1985. Our success is contributed to good immunization coverage and an enhanced AFP surveillance system. The Institute for Medical Research (IMR), Malaysia, was designated the National Poliovirus Laboratory (NPL) in 1992. Since then, our polio laboratory has collaborated actively with the Disease Control Division, Ministry of Health (MOH), and WHO towards achieving polio eradication. The polio laboratory plays an important role in surveillance with the virological investigation of AFP cases. The objectives of this paper are to review isolation of Sabin polioviruses and non-polioviruses before and after switch of OPV to IPV in the childhood immunization schedule and share experiences in the performance of the national reference laboratory.

## 2. Materials and Methods

### 2.1. Samples

Since 1992, when the Virology Unit, IMR, was designated as the NPL, till December 2012, the NPL received 3360 stool specimens from 1930 reported AFP cases sent from hospitals throughout Malaysia. Another 619 specimens, comprised of throat swabs, and cerebrospinal fluid were also received from these cases. The specimens were accompanied by an AFP notification form with details of patient personal and clinical history.

### 2.2. Virus Isolation Using WHO Standard Protocol

Till 2008, the methods used for virus isolation and microneutralization for identification of positive isolates were as described in WHO Polio Laboratory Manual 2004 and, from 2008 onwards, the supplemental manual of 2006 for the New Algorithm Technique was used for poliovirus isolation. All stool specimens were processed with chloroform before inoculation into RD and L20B cell lines from our laboratory stock held in liquid nitrogen at low passage. Inoculated cell cultures were examined daily for cytopathological effect (CPE) and confirmed by microneutralization assay using standard WHO antisera. Poliovirus isolates were sent to the Victorian Infectious Disease Reference Laboratory (VIDRL) in Melbourne, Australia, for further identification and intratypic differentiation (ITD), till August 2010.

Between 2008 and 2010, following a new WHO standard algorithm, all positive cultures in L20B were sent to the VIDRL in Australia for ITD and microneutralization assay was not done.

### 2.3. WHO Standard Algorithm Used after August 2010 Poliovirus Diagnostic Real-Time Reverse Transcriptase Polymerase Chain Reaction (rRT-PCR) Assay

The method for ITD of poliovirus isolates by rRT-PCR was as recommended by WHO using kit developed by USA Centers for Disease Control and Prevention.

The principles of the rRT-PCR technique involved the conversion of viral RNA (vRNA) to complementary DNA (cDNA) using reverse transcriptase. The cDNA was amplified in a PCR reaction using Taq polymerase and simultaneously the PCR products were detected by specific TaqMan probes. Both the cDNA synthesis and the PCR reaction used multiple sets of oligonucleotide 6 primers (PV serotype 1, PV serotype 2, and PV serotype 3, pan-poliovirus, pan-enterovirus, and Sabin multiplex) and probes. This combination of primers and probes resulted in serotype identification and intratypic differentiation of poliovirus isolates. Positive- and negative-control reactions were included in each set of extractions.

Specimens that were diagnosed as Sabin-like were further screened for VDPV using a VDPV diagnostic rRT-PCR kit. The primers and probes used consisted of Sabin 1 VDPV, Sabin 2 VDPV, and Sabin 3 VDPV. Specimens that were diagnosed as group-specific for enterovirus (pan-enterovirus-positive) but negative for polioviruses were further sequenced for non-polio enterovirus differentiation.

### 2.4. AFP Clinical Picture

All AFP reported cases were followed up for 60 days to ascertain residual paralysis. This activity was monitored by the Surveillance Unit, Disease Control Division of the Ministry of Health, by communication with clinicians attending to the AFP cases and from monthly notification data obtained from State Health Departments. Clinical assessment of all reported AFP cases was also reviewed at the Expert Polio Review Meetings.

## 3. Results

One hundred and seventy-six enteroviruses were isolated from the 3360 stool specimens investigated between 1992 and December 2012 (Table 1). 55 out of 176 virus isolates were polioviruses (PV) and the remaining were non-polio enteroviruses (NPEV). Since 2009, after the sequential introduction of IPV in the childhood immunization programme, no Sabin polioviruses were isolated. Out of 55 polioviruses isolated, 3 were wild type isolated in 1992, caused by importation of wild polioviruses that originated from the Indian subcontinent [6]. The wild-type polioviruses were confirmed by intratypic differentiation by Centers for Disease Control, Atlanta, USA. The remaining were vaccine-related Sabin-like strains. Out of these, 21 were Sabin type 3 viruses, 15 were Sabin type 2, 7 were a mixture of Sabin 2 and Sabin 3, and 9 were Sabin type 1.

**Figure 1:**
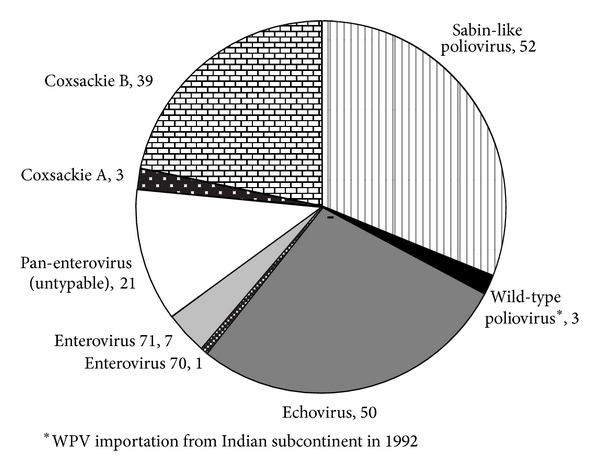
Polioviruses and non-polio enteroviruses isolated from AFP cases from 1992 till 2012.

The non-polio enteroviruses (NPEV) included coxsackie A viruses, coxsackie B viruses, echoviruses, and enterovirus 71. Some of the isolates were untypable (Figure 1).

## 4. Discussion

The primary goal of the AFP surveillance for poliovirus eradication is to promptly detect possible areas of circulating wild poliovirus and circulating vaccine-derived poliovirus to implement immediate control measures. It remains essential that surveillance is maintained until global eradication is achieved because of the risk of wild virus importation from endemic regions.

In Malaysia, the last major outbreak of poliomyelitis occurred in 1977 with 121 cases including 4 deaths [16]. The number of poliomyelitis cases decreased dramatically from 1978 following an effective National Oral Polio Vaccine Immunization Programme introduced in 1972. However, three cases of poliomyelitis were reported in 1992 due to importation of wild poliovirus [17]. Since 1993, no wild poliovirus has been identified. Malaysia together with other countries in the Western Pacific Region (WPR) was certified as polio-free in 2000. Since then, considering the reduced risk of wild-type poliovirus indigenous or imported, many countries including Malaysia had started using IPV either completely or sequentially, driven by concerns to avoid VAPP. The National Committee on Immunisation Policy and Practice (NCIPP), Ministry of Health, reviewed the safety, immunogenicity, herd immunity, cost benefit, and practicality such as implementation issues and sustainability. The transition policy in Malaysia from OPV to IPV began in 2008 and by January 2010 was implemented in all states. For overall public health benefit, and based on recommendations by the WHO, a sequential vaccination schedule of 4 doses of IPV is given to infants at the second, third, and fifth months of age followed by a booster dose at 18 months. A second booster with OPV is given at 7 years of age.

The challenge in the use of IPV is the vigilance of WPV importation from wild polio-endemic areas. As long as WPV transmission has not been interrupted everywhere, all polio-free countries and areas remain at risk of reimportation of WPV, particularly from the remaining polio-endemic countries. From 2003 to 2009, WHO has recorded 133 WPV importation events in 29 previously polio-free countries [18]. The risk of importations with subsequent spread was the highest in countries immediately bordering endemic countries and was also higher in countries with low routine immunization coverage. In 2011, WPR experienced a wild poliovirus importation from Pakistan into western China, causing a polio outbreak of 21 cases in young children and adults in Xinjiang Uyghur Autonomous Region [19]. The outbreak in China reaffirms the continued risk for any country to be reinfected until such time as all wild poliovirus transmission is interrupted globally.

In May 2012, at the World Health Assembly, the WHO's Executive Board (EB) adopted a landmark resolution, declaring the completion of polio eradication a “programmatic emergency for global public health,” requiring the full implementation of current and new eradication strategies [20]. This includes maintaining very high population immunity against polioviruses through routine immunization programmes, keeping vigilance for poliovirus importations, and the emergence of cVDPVs, by achieving and sustaining certification-standard surveillance for polioviruses. Timely identification of polioviruses and VDPVs that can cause acute flaccid paralysis (AFP) is becoming increasingly important because of reported circulating-VDPV outbreaks.

In this respect, the NPL in IMR has continuously strengthened the laboratory AFP surveillance with monthly reporting of the virological investigation of AFP cases to WHO and to the Ministry of Health, AFP Review Committee. The performance of NPL is annually reviewed by WHO-WPRO using various quality indicators such as accuracy and timeliness of results. The annual performance review assessment also includes an on-site laboratory inspection by an expert virologist and proficiency testing of panel specimens. The NPL has achieved these quality indicators set by WHO to reach international standards required for certification of poliomyelitis eradication as evidenced by the accreditation status awarded by the WHO in the Global Polio Laboratory Network since 1998. When the new standard WHO algorithm for poliovirus isolation and identification was introduced in the WPR laboratory network in 2006, our NPL introduced the algorithm for virus isolation in 2008 and real-time polymerase chain reaction method was used for poliovirus ITD in 2010. Only discordant results are sent for verification to the RRL in Australia. This is important for prompt investigation of poliovirus variants isolated for any potential outbreak management.

Fifty-five polioviruses were isolated during the 20-year period from 1992 till 2012. Except for the 3 cases of wild poliovirus importation from the Indian subcontinent, the remaining were Sabin-like strains. No VDPV has been isolated, attributed possibly to Malaysia's high immunization coverage. Since the sequential introduction of IPV in the EPI, no Sabin polioviruses were isolated from AFP cases. However, 26 Sabin polioviruses were isolated from non-AFP cases in the laboratory between 2009 and 2012 [20]. In 2012, the laboratory AFP surveillance was supplemented with environmental surveillance and sewage sampling. Thirteen Sabin polioviruses were also isolated from sewage in the same year, but no VDPV was detected [20]. With near eradication of poliomyelitis, viruses other than polioviruses have been reported to cause AFP [21]. Among these viruses are echoviruses, coxsackie viruses, and enterovirus 71 which are also members of the* Enterovirus *genus and associated with a vast range of clinical presentations, from asymptomatic, hand-foot-mouth disease to acute flaccid paralysis resembling polio. One hundred and twenty-one NPEV were isolated between 1992 and 2012. The NPEV isolated belonged to the group coxsackie A viruses, coxsackie B viruses, echoviruses, and enterovirus 71. Some of the isolates were untypable. During the past decade, the NPEV isolation rate has been low. The laboratory has reviewed and investigated many contributing factors but the isolation rate has been sustained below 3%, possibly attributed to good public health and hygiene. Seven out of 121 NPEVs were enterovirus 71 and the clinical picture for these cases included acute encephalopathy and enteroviral monoplegia. EV 71 has been reported in countries of the Asia Pacific Region causing large outbreaks of hand-foot-mouth disease (HFMD). In Malaysia, the first major HFMD outbreak occurred in 1997 and in 2000, where EV71 was reported to cause neurological complications in some children which included acute flaccid paralysis and brain stem encephalitis [22]. Since then, several HFMD outbreaks have occurred in cyclic patterns, complicated by fatalities due to severe neurological involvement; EV71 has been implicated as the major causative agent for these outbreaks [23].

## 5. Conclusion

A high level of laboratory surveillance has shown that no outbreaks of wild poliovirus have occurred since 1992. No cases of VDPV were recorded. The NPL continues to play an integral role in the AFP surveillance and is committed towards WHO's goal of polio eradication.

## Figures and Tables

**Table 1:** AFP isolates between 1992 and July 2012.

Year	Number of AFP cases	Number of stool specimens	Enterovirus isolate
1992	10	14	3 PV, 1 NPEV
1993	15	28	1 PV, 6 NPEV
1994	16	28	1 NPEV
1995	17	30	4 PV, 6 NPEV
1996	32	44	3 PV, 2 NPEV
1997	77	118	6 PV, 6 NPEV
1998	86	147	4 PV, 4 NPEV
1999	85	130	4 NPEV
2000	147	250	6 PV, 18 NPEV
2001	108	146	3 PV, 19 NPEV
2002	58	94	5 PV, 4 NPEV
2003	97	150	7 PV, 10 NPEV
2004	128	194	9 NPEV
2005	146	283	4 PV, 5 NPEV
2006	118	240	4 PV, 4 NPEV
2007	110	220	0 PV, 6 NPEV
2008	131	229	5 PV, 1 NPEV
2009	106	199	1 NPEV
2010	142	250	3 NPEV
2011	143	252	7 NPEV
2012	158	314	6 NPEV

Total	1930	3360	176
